# Perinatal Identification, Referral, and Integrated Management for Improving Depression: Development, Feasibility and Pilot Randomised Controlled Trial of the PIRIMID System

**DOI:** 10.3390/healthcare13202578

**Published:** 2025-10-14

**Authors:** Charlene Holt, Sarah Maher, Alan W. Gemmill, Lauren A. Booker, Sabine Braat, Digsu N. Koye, Bianca Pani, Anne Buist, Jeannette Milgrom

**Affiliations:** 1 Parent-Infant Research Institute, Austin Health, Melbourne, VIC 3081, Australiaalan.gemmill@austin.org.au (A.W.G.); l.booker@latrobe.edu.au (L.A.B.); bianca.pani@austin.org.au (B.P.); jeannette.milgrom@austin.org.au (J.M.); 2 Institute for Breathing and Sleep, Austin Health, Heidelberg, VIC 3084, Australia; 3 Violet Vines Marshman Centre for Rural Health Research, La Trobe Rural Health School, La Trobe University, Bendigo, VIC 3552, Australia; 4 Centre for Epidemiology and Biostatistics, Melbourne School of Population and Global Health, University of Melbourne, Melbourne, VIC 3053, Australia; s.braat@unimelb.edu.au (S.B.); digsu.koye@unimelb.edu.au (D.N.K.); 5 Methods and Implementation Support for Clinical and Health (MISCH) Research Hub, Faculty of Medicine, Dentistry and Health Sciences, University of Melbourne, Melbourne, VIC 3053, Australia; 6 Department of Psychiatry, University of Melbourne, Parkville, VIC 3052, Australia; a.buist@unimelb.edu.au; 7 Melbourne School of Psychological Sciences, University of Melbourne, Parkville, VIC 3010, Australia

**Keywords:** postnatal depression, screening, clinical decision support, treatment uptake, referral pathways, randomised controlled trial

## Abstract

**Background/Objectives**: Postnatal depression imposes a substantial burden on wellbeing as well as costs estimated to exceed $7 billion for every one-year cohort of births in Australia. Despite this, most cases go untreated, a major barrier being the poor rate of treatment uptake. We developed and pilot tested an integrated screening and clinical decision support system (PIRIMID) to assist maternal and child health nurses (MCHNs) to create individualised management plans, with specific referral pathways, for women depressed postnatally. We assessed the feasibility of PIRIMID by examining acceptability for both nurses and women, ease of implementation, and referral rates, and we monitored treatment uptake and depression. **Methods**: An extensive co-design and consultation process was used to develop PIRIMID. A pilot cluster randomised controlled trial (RCT) was conducted comparing PIRIMID to Routine care, with partial crossover (PIRIMID followed by crossover to Routine care and Routine care followed by continued Routine care). A state-wide survey of MCHNs in Victoria, Australia, explored perceived benefits and barriers of PIRIMID from a consumer perspective. **Results**: Twelve MCHNs (PIRIMID: *n* = 6; Routine care: *n* = 6) and 229 women (conditions: PIRIMID, *n* = 52; Crossover Routine care, *n* = 42; Routine care, *n* = 57; Continued Routine care, *n* = 78) were recruited to the RCT. Median scores for depression, anxiety and stress symptoms were low and similar at all timepoints and conditions. PIRIMID was acceptable and helpful to MCHNs and women, and most MCHNs rated integration into their existing clinical systems as easy. There were trends in favour of higher referral rates by PIRIMID MCHNs (18%, 95% CI: 5–40) compared with other conditions (10–15%, 95% CIs: 6–29, 2–27, 6–26), but treatment uptake appeared similar across conditions. The statewide survey (*n* = 292) revealed that 84% of MCHNs would use PIRIMID, and the main potential barriers to use would be time constraints and technical issues. **Conclusions**: This pilot work indicates that PIRIMID shows promise as a feasible and acceptable tool to assist MCHNs to develop management plans for women depressed postnatally. Further research with adequate statistical power is needed to explore effects on treatment uptake with larger samples of postnatally depressed women.

## 1. Introduction

In the first postnatal year, between 10% and 17% of mothers will experience depression [1,2,3] and approximately 15% will experience anxiety [4]. Prevalence estimates vary depending on the country and population characteristics, the psychometric instruments used (screening questionnaires compared to diagnostic interviews), and whether period or point prevalence estimates are reported. For example, a meta-analysis of studies using only diagnostic interviews [5] found the period prevalence within the first year post-birth was 7% for Major Depressive Disorder and 12.1% for all depression. A systematic review of reviews [6] reported a higher estimate for self-report measures (27.4%) than for structured interviews (17%).

Postnatal depression (PND) impacts not only a woman’s physical, emotional and mental wellbeing, but also her relationships with her partner and infant [7]. This can have significant consequences for the infant, including impaired mother-infant interactions, which are crucial to optimal development, and may have long-term negative effects on child development [8].

PND has a high economic burden on society [9]. In Australia, it is estimated that the cost of perinatal mental health problems exceeds $7 billion for every one-year cohort of births, largely attributable to the lifetime impacts on children [10].

### 1.1. Universal Screening

Although universal screening for PND has shown increased rates of referral and subsequent engagement with treatment, as well as some improvements in maternal mental health [11], treatment uptake remains suboptimal. A recent meta-analysis showed that among women who screened positive for perinatal depression, only 55% accessed treatment [12]. Even fewer women (around 6–10%) receive an adequate dose of treatment [13,14].

Effective integration of universal screening and pathways to care is essential for improving outcomes [14]. However, primary care services often lack integrated systems to ensure best-practice, consistent, efficient identification and appropriate management [15]. In addition, they lack on-the-spot gold-standard guidance for interpreting, and acting upon, positive depression screening results. In practice, time-constrained health professionals are required to integrate information relevant to the woman’s presentation and make referral decisions [15].

If universal screening is supplemented with guidance in the decision-making process, potential mismatches between screening scores and treatment provided could be avoided. When health professionals have access to appropriate decision-making guidance, such as using a clinical decision support system, they make more informed decisions [16].

Torti and colleagues [17] developed a toolkit to standardise screening, referral and education for perinatal mental health disorders that consisted of screening forms, a referral algorithm, resource list, information for patients, and educational materials for staff. Although the toolkit resulted in improved screening and referral rates [17], evidence regarding its impact on treatment uptake and depression symptoms is lacking.

Universal screening approaches that have shown benefits for depression symptoms often involve comprehensive training, support, and screening algorithms with treatment pathways [18]. Others use intensive systems of care with multidisciplinary dedicated teams reviewing all mothers with a positive screening result [19]; however, this approach is resource-intensive and costly. Approaches are most likely to be successful when they integrate screening and treatment and are delivered by a health professional known to the mother. There is increasing interest in developing more cost-effective decision-making tools.

### 1.2. Electronic Clinical Decision Support Systems

Recently, electronic clinical decision support systems (CDSSs) have been developed for depression management in the general population. They are designed to improve healthcare by aiding in decision-making using specialised clinical knowledge, patient data, and other health-related information [20]. There is evidence that they can successfully increase uptake of treatment [21] and improve depression symptoms [22].

A common CDSS approach involves creating predetermined score-based categories that guide corresponding treatment recommendations. For example, an Australian study developed a novel electronic clinical prediction tool to screen patients for depression and group them into low, medium and high risk of ongoing depression and stratify them into different treatments [23,24]. The tool was shown to be clinically effective, with reduced depression symptoms compared to a control group at three months [22].

To our knowledge, only one electronic CDSS for PND has been described in the literature. The Maternity Case-finding Help Assessment Tool (MatCHAT) screens women electronically using the Patient Health Questionnaire-2 (PHQ-2) and a positive screen triggers further screening with the PHQ-9 [25]. Women are also screened for anxiety, family violence and substance use. An accompanying resource booklet provides referral information (including referral letter templates) categorised by symptom severity. In a feasibility study with 3 midwives and 20 mothers, MatCHAT was found to be useful and acceptable [25]. However, uptake of the tool by midwives was low with 25 invited to participate, 10 expressing interest, and only 3 using the tool. Potential barriers to implementation identified in the study included time constraints, concerns that screening might uncover complex follow-up needs, limited Wi-Fi access, and the challenges of working across multiple locations.

### 1.3. The PIRIMID System

This project aimed to develop, assess feasibility and pilot an electronic CDSS for PND called ‘Perinatal Identification, Referral and Integrated Management for Improving Depression’ (PIRIMID). PIRIMID is an innovative advance over existing e-screening and symptom-based referral systems by incorporating case formulation principles to guide maternal and child health nurses (MCHNs) in developing management plans tailored to each woman’s specific needs. In contrast to typical approaches, which categorise women based solely on screening scores and suggest referral options or support resources accordingly, PIRIMID provides structured guidance that considers the individual context and complexities of the woman’s situation. The system offers immediate best practice guidance for interpreting and responding to positive depression screening results, adhering to national Clinical Practice Guidelines [26]. As noted by Wright and colleagues [25], health professionals may feel apprehensive about uncovering complex issues; PIRIMID offers prompts and guidance to support their confidence. Designed to be time-efficient, PIRIMID guides health professionals through four steps that generate a personalized management plan during the consultation with the woman. The system uses check boxes to streamline entry of information, and MCHNs are trained to concisely capture the core of the woman’s difficulties. Scores are automatically calculated and flagged, where appropriate. The management plan can be saved in clinical notes or used for referral. The system is fully electronic, eliminating the inefficiencies of combining digital and paper-based materials, supporting smoother integration into clinical practice.

PIRIMID begins with e-screening using the Edinburgh Postnatal Depression Scale (EPDS) [27], addressing the limitations of paper-and-pencil screening, which is often inconsistently applied and prone to error [28]. It also includes the Whooley case-finding questions [29] and screens for psychosocial risk factors for PND, including personal or family history of mental health problems, antenatal emotional difficulties, major life events, limited practical or emotional support, lack of partner support, and feeling unsafe. Development of PIRIMID was guided by the framework described by Medlock and colleagues [16], which considers both health professional factors (e.g., required clinical knowledge, types and wording of clinical advice) and patient factors (e.g., relevant attributes and data).

**Figure 1:**
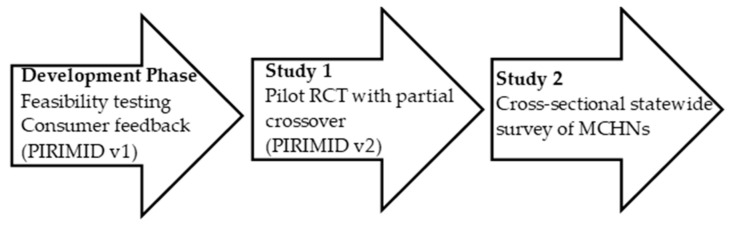
Stages in the development of the PIRIMID system.

This project consisted of a development phase followed by two studies. The development phase included feasibility testing comparing e-screening to traditional paper-and-pencil screening, followed by consumer feedback from MCHNs testing the prototype PIRIMID system. Next, Study 1 aimed to evaluate the PIRIMID system (Version 2) against Routine care provided by MCHNs in a pilot cluster randomised controlled trial (RCT) to evaluate feasibility and inform a future confirmatory RCT. Maternal outcomes were (a) rates of referral and treatment uptake; (b) depression, anxiety and stress; and (c) acceptability. MCHN outcomes were (a) rates of screening; (b) staff time; and (c) acceptability. The primary research question was whether the PIRIMID system is a feasible and acceptable tool for MCHNs and postnatal women. Study 2 was a cross-sectional statewide survey of MCHNs that aimed to understand the perceived benefits and barriers of the PIRIMID system. Figure 1 provides a visual overview of the development process used in this project.

## 2. Methods

### 2.1. Development of the PIRIMID System and Feasibility

#### 2.1.1. Conceptualisation

The PIRIMID system was developed consistent with international guidelines for perinatal mental health both in Australia (‘Mental Health Care in the Perinatal Period Australian Clinical Practice Guidelines’) [26] and the United Kingdom (National Institute for Health and Care Excellence (NICE) ‘Antenatal and Postnatal Mental Health: Clinical Management and Service Guidance’) [30], for use by primary care professionals, such as MCHNs. In the early postnatal months, MCHNs in Victoria, Australia, offer ten free consultations to families with a new baby and play a pivotal role in the early detection of PND as the primary providers of screening.

#### 2.1.2. Description of the PIRIMID System

PIRIMID is a web-based platform available on the MCHN’s desktop as an interactive dashboard. e-Screening was completed on a tablet and scores automatically imported into PIRIMID, including total EPDS score, EPDS anxiety sub-score, EPDS self-harm (item #10) score, response to Whooley questions, and endorsed risk factors. Explanation and interpretation for each score, along with recommended actions, are provided. PIRIMID is structured around four steps:

Step 1: The MCHN identifies and marks scores above threshold (flagged by PIRIMID) and relevant to the woman’s presentation.

Step 2: The MCHN records a brief summary of the woman’s presenting concerns, history and pertinent clinical information.

Step 3: The MCHN integrates screening results with contextual and other relevant information to develop a clinical formulation.

Step 4: PIRIMID presents referral pathways and follow-up options, allowing the MCHN to develop a management plan in consultation with the woman. PIRIMID consolidates information from Steps 1–4 into a printable, one-page plan that can be saved in clinical notes or used for referral to a general practitioner.

#### 2.1.3. Feasibility and Iterations of the PIRIMID System

The development phase included feasibility testing by MCHNs in the City of Whittlesea, Victoria, Australia, during routine 4-week Key Ages and Stages (KAS) visits, when they assess each woman’s emotional health.

First, three MCHNs trialled e-screening while three others continued their usual practice (June 2016–August 2017). This established that recruitment of MCHNs and women (*n* = 72) was feasible, and e-screening was comparably acceptable with traditional paper and pencil screening. Women rated the assessment’s helpfulness and their comfort discussing emotional health on a 10-point Likert scale (1 = unhelpful/uncomfortable; 10 = extremely helpful/comfortable). For helpfulness: e-screening, Median (IQR) = 10 (8–10), *n* = 28; Paper & pencil, 8 (6–10), *n* = 39. For comfort: e-screening, 10 (9–10), *n* = 29; Paper & pencil 10 (9–10), *n* = 41.

Next, two MCHNs provided feedback via interview on the prototype PIRIMID system following use in their practice (*n* = 24 women; August 2017–February 2018). Based on this feedback, the screening results summary and flagged items were revised, labels reworded for clarity, and the management plan’s format and content enhanced, resulting in version 2 of PIRIMID.

Version 2 of PIRIMID was then tested in the pilot RCT (Study 1 below), and MCHNs who participated provided feedback via focus groups. Feedback highlighted that the most useful features were automatic scoring, result interpretations, the prompts and reassurance that their interpretations were correct. Reported challenges included time constraints and duplicating information already in client records. Suggestions also addressed wording, flow, and visibility of EPDS item responses. Redundant features were thus removed and further improvements made to the layout, navigation and readability, resulting in version 3 of the PIRIMID system.

### 2.2. Study 1: PIRIMID Versus Routine Care: Randomized Controlled Trial with Partial Crossover

#### 2.2.1. Trial Design

A parallel two-arm cluster RCT with partial crossover was implemented from July 2019–December 2021, comparing PIRIMID to routine care (Australian New Zealand Clinical Trials Registry no: ACTRN12616001217493, Registered on 2 September 2016). Twelve MCHNs were randomly allocated to one of two conditions: PIRIMID system (*n* = 6) or Routine care (*n* = 6).

In the first 12 months of the RCT (referred to as study period 1), MCHNs were asked to enrol as many women as possible. After 12 months, the MCHNs in the PIRIMID condition crossed over to the Routine care condition and continued enrolling women and providing routine care for an additional 18 months (referred to as study period 2). The MCHNs who were randomly allocated to the Routine care condition provided routine care during study periods 1 and 2. Maternal outcomes were rates of referral and treatment uptake, and depression, anxiety, and stress symptom severity at follow-up (3 months post-birth). MCHN outcomes were rates of screening and staff time. Mothers and MCHNs provided feedback on acceptability of the system. This trial was approved by the Human Research Ethics Committee of Austin Health on 19 April 2016 (Project no. HREC/15/Austin/273).

#### 2.2.2. Participants

Twelve MCHNs from the City of Whittlesea Maternal and Child Health service in Victoria, Australia, were recruited in pairs matched for level of experience (greater or less than 5 years) to ensure balance between the two conditions. MCHNs invited women attending the 4-week post-birth KAS visit to participate in the study. Women were provided with a Participant Information and Consent Form to read and sign.

#### 2.2.3. Eligibility Criteria

Inclusion criteria were women >18 years of age with a baby aged 4–6 weeks and ability to understand spoken and written English. MCHNs were included if they routinely conducted 4-week KAS visits.

#### 2.2.4. Interventions

PIRIMID. The PIRIMID system (v2) was used to complete the emotional health assessment with all women at the 4-week KAS visit. Prior to recruitment, MCHNs in the PIRIMID condition attended a 1.5-h group training session on the PIRIMID system, delivered by two clinical psychologists and the project manager. During study period 2, the MCHNs no longer had access to the PIRIMID system.

Routine care. Current best practice for MCHNs is to complete an emotional health assessment with all women at the 4-week KAS visit. This consisted of screening women with the EPDS in paper-and-pencil format. Scores ≥ 13 indicate high likelihood of PND.

#### 2.2.5. Measures

Baseline questionnaires (including demographic information) were completed by women at the end of the 4-week KAS visit initially by paper and pencil, and then electronically using the Qualtrics platform from 2020 onwards due to the COVID-19 pandemic. Follow-up questionnaires were completed using Qualtrics at 3 months post-birth. Women who did not complete questionnaires electronically were invited to complete them by telephone. At each 4-week KAS visit, MCHNs completed a log of all women seen for a 4-week KAS visit. MCHNs in the PRIIMID condition also completed a feedback form at study end. Table 1 shows outcomes and data collection time points.

#### 2.2.6. Maternal Outcome Measures

Referral rates. MCHNs recorded in their log referrals made to other health professionals that were for support of the woman’s emotional well-being.

Treatment uptake. At the 4-week KAS visit, women were asked whether they sought help since baby’s birth for feelings of depression, anxiety, stress, sleeping difficulties, marital/relationship difficulties, financial difficulties, feeling isolated, overwhelmed by routine, as advised by midwife or doctor, or for other reasons (yes/no). The 3-month post-birth questionnaire asked women which services or programs they accessed to manage their mood since their 4-week post-birth appointment with their nurse (yes/no).

Depression, Anxiety and Stress. These were measured using the short form of the Depression Anxiety Stress Scales (DASS-21), using the recommended cut-offs for severity ratings: ‘moderate to severe depression’ (scores ≥ 14), ‘moderate to severe anxiety’ (scores ≥ 10), ‘moderate to severe stress’ (scores ≥ 19) [31].

Depressive Disorder. Women were assessed with the Structured Clinical Interview for DSM-5 (SCID-5) [32] by a clinical psychologist blind to allocation to identify presence of a clinical depressive disorder.

Acceptability to women. Women were asked: “Was the assessment of your emotional health helpful?” and “How comfortable did you feel discussing your emotional health with your nurse?” Responses were on a 10-point Likert scale (1 = unhelpful/uncomfortable and 10 = extremely helpful/extremely comfortable).

#### 2.2.7. MCHN Outcomes Measures

Screening rates. MCHNs recorded in their log whether each woman was screened with the EPDS, Whooley questions, and for psychosocial risk factors.

Staff time. MCHNs recorded in their log the duration of each KAS visit. PIRIMID MCHNs were also asked, “Was using the CDSS a more or less time-consuming way of developing a management plan?” Responses were on a 10-point Likert scale (1 = more time-consuming and 10 = less time-consuming).

Acceptability to MCHNs. MCHNs in the PIRIMID condition were asked, “How helpful did you find the PIRIMID tool?” and “How easily could the CDSS be integrated into your existing clinical practice?” Responses were on a 10-point Likert scale (1 = unhelpful/difficult and 10 = extremely helpful/easy). They also completed an adapted version of the System Usability Scale (SUS) [33], which included ten items asking them to rate their level of agreement (1 = Strongly Disagree to 5 = Strongly Agree) with positive and negative statements about the system (e.g., “I thought the Clinical Decision Support System was easy to use”). A score of 68 on the SUS is average and a score above 80 is excellent.

#### 2.2.8. Sample Size

Initially, this study was designed as a parallel two-arm cluster RCT with 40 clusters (MCHNs) each recruiting 15 postnatal women to yield a sample size of *n* = 600. The sample size was calculated based on our previous experience [34] that recruiting and screening 50 women per month for 12 months across 40 MCHNs would be feasible. With 80% power at two-sided α = 0.05, considering a conservative estimate of the intra-cluster correlation (0.1) with a cluster size of 15 women per nurse, the power achieved with *n* = 600 would be sufficient to detect small-to-medium effect sizes, as categorised by Cohen [35].

However, the trial was impacted by the COVID-19 pandemic and associated restrictions. Recruitment of MCHNs and postnatal women was slower than anticipated. After 12 months, only 12 MCHNs had been recruited, and projections indicated that the original target sample size of MCHNs and women would not be feasible. To address this, the study investigators ceased recruitment of MCHNs and amended the study design to be a partial crossover. This provided the practical advantage of having the PIRIMID-trained MCHNs revert to paper and pencil as a real-world comparison of whether any learnings from PIRIMID persisted. The Routine care MCHNs continued their usual care, preserving a useful comparison group. Recruitment to the trial ceased once the funding was exhausted. This modification to the trial allowed for meaningful comparisons, despite a reduced sample size, and provided real-world insights.

#### 2.2.9. Randomisation

A computer-generated permuted blocks (block size of 2) randomised allocation schedule produced by an independent researcher and administered using sealed opaque envelopes by administrative staff not involved in the project was used to assign MCHNs to either condition using a 1:1 allocation ratio. Author SM provided the administrative staff member with the names of MCHNs to be enrolled in the study, and the administrative staff member sequentially allocated an ID number to each name and provided the researcher with the sealed opaque envelope containing the allocation. To help ensure comparability of MCHNs in each condition, the randomised allocation schedule was stratified by years of professional experience (greater or less than 5 years).

#### 2.2.10. Blinding

The administrative staff involved in randomisation and the clinical psychologists administering the SCID-5 were blind to allocation. Given the nature of the intervention, MCHNs and women could not be blinded beyond allocation.

#### 2.2.11. Statistical Methods

Due to the modest sample size, only descriptive statistics were performed. The most appropriate descriptive statistic for the distribution of the data was reported (mean ± standard deviation, frequency and %, median and interquartile range [25th to 75th percentile] or range [minimum-maximum]). Two-sided 95% Clopper-Pearson confidence interval were provided for the primary pilot outcome, referral rates. Statistical analyses were performed using IBM SPSS Statistics v 30.0 (SPSS Inc., Chicago, IL, USA).

### 2.3. Study 2: Statewide Survey of MCHNs

#### 2.3.1. Study Design and Participants

This was a cross-sectional survey of MCHNs in all municipalities across Victoria, Australia (Austin Health Human Research Ethics Committee no: HREC/15/Austin/273).

#### 2.3.2. Procedure

An email and reminder were sent to MCHN co-ordinators either directly or via their local council with information and a link to an anonymous online survey (hosted on Qualtrics). MCHN coordinators were asked to circulate the link to their staff.

#### 2.3.3. Survey

A survey was developed to explore MCHNs’ perceptions of barriers and facilitators to using a PND screening and clinical decision support system. Items asked MCHNs: (1) how they assess clients’ emotional well-being at the 4-week KAS visit; (2) satisfaction with this method (5-point Likert scale, 1 = very dissatisfied and 5 = very satisfied); (3) confidence in responding when a woman scores above the EPDS threshold (5-point Likert scale, 1 = not at all confident and 5 = very confident); (4) willingness to use an online system such as PIRIMID (yes/no/unsure); (5) perceived benefits of PIRIMID (free text); (6) potential barriers to use (free text); (7) factors that would facilitate use (free text); and (8) suggestions for improving depression and anxiety screening (free text).

#### 2.3.4. Statistical Methods

For quantitative data, as for Study 1, the most appropriate descriptive statistic for the distribution of the data was reported. Analyses were performed using IBM SPSS Statistics v30.0 (SPSS Inc., Chicago, IL, USA). Text responses from open-ended questions were analysed using conceptual content analysis. This was done by hand by one coder. Initial common categories were identified and then coded, with flexibility allowed to add categories throughout the coding process. General trends and patterns were identified.

## 3. Results

### 3.1. Study 1: PIRIMID Versus Routine Care: Randomized Controlled Trial with Partial Crossover

#### 3.1.1. Participant Flow

**Figure 2:**
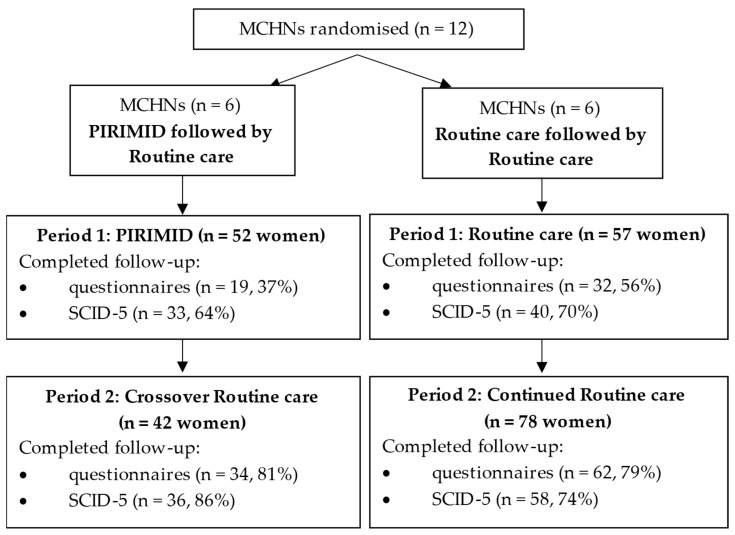
Number of women recruited in each condition across the two study periods MCHN = Maternal and Child Health Nurse; PIRIMID = Perinatal Identification, Referral and Integrated Management for Improving Depression; SCID-5 = Structured Clinical Interview for the DSM-5. Note: Study periods 1 and 2 did not include the same women.

Figure 2 presents recruitment and loss to follow-up by condition across the two study periods of the RCT. Recruitment and data collection occurred between July 2019 and March 2022. Questionnaire completion was low in study period 1, but improved in study period 2 following changes to data collection (collected electronically and option to complete by telephone).

#### 3.1.2. Baseline Characteristics

Table 2 shows the baseline characteristics of participants, which are relatively similar across the conditions and time periods; however, there appears to be fewer Australian-born women in the PIRIMID condition compared to Routine care. Cluster sizes are also shown in Table 2. Of the 6 MCHNs in the PIRIMID followed by Routine care condition, five were female (83%) and one was male (17%). All MCHNs in the Routine care followed by Routine care condition were female (100%). In both conditions, MCHNs were equally divided on years of experience (*n* = 3 with ≤5 and *n* = 3 with >5 years).

#### 3.1.3. Maternal Outcomes

Table 3 shows rates of referral by MCHNs at the 4-week KAS visit and treatment uptake by women, both prior to the 4-week KAS visit and between the 4-week KAS visit and 3-month follow-up. Referral rates appear slightly higher in the PIRIMID condition; however, treatment uptake is similar across the conditions and study periods.

Depression, anxiety and stress outcomes are also shown in Table 3, along with depressive disorder diagnoses at 3-months follow-up. Overall, median scores were low for all conditions on depression, anxiety and stress symptoms at all timepoints. The proportion of women scoring in the ‘moderate-severe’ range on the DASS depression, anxiety, and stress scales was below 20%.

Descriptive differences were observed in the proportion of cases with ‘moderate-severe’ depression and anxiety between the 4-week KAS visit and 3-month follow-up in the PIRIMID versus Routine care conditions (see Table 3). In study period 1, among women with complete data, ‘moderate to severe’ depression was reported by 11% (2/19) at 4-weeks and 5% (1/19) at 3 months in the PIRIMID condition, compared to 13% (4/31) and 19% (6/31) in Routine care. For anxiety, rates were 16% (3/19) at 4 weeks and 0% (0/19) at 3 months in PIRIMID, compared with 10% (3/31) at both time points in Routine care. For stress, rates were 11% (2/19) at both timepoints in PIRIMID and 16% (5/31) at both timepoints in Routine care.

As shown in Table 3, women in both conditions reported high acceptability of the emotional health assessment at the 4-week KAS visit, with median helpfulness and comfort scores of 8–10.

#### 3.1.4. MCHN Outcomes

Table 4 shows high screening rates with the EPDS across conditions and study periods (100%). Screening using the Whooley questions (98%) and psychosocial risk factors (100%) was highest in the PIRIMID condition during study period 1. As shown in Table 4, most 4-week KAS visits in the PIRIMID condition during study period 1 (91%, 19/21) took the standard 45 min. However, 5 of 6 MCHNs rated PIRIMID as more time-consuming for developing a management plan (score 1–5), while 1 of 6 rated it as less time-consuming (score 6–10).

MCHNs provided positive feedback on PIRIMID, rating its helpfulness as 5 (*n* = 1, 17%), 7 (*n* = 2, 33%) and 8 (*n* = 3, 50%). System Usability Scale scores were slightly below average (median = 60, range = 35–83). Most MCHNs (5/6) rated integration of PIRIMID into existing clinical systems as easy (score 6–10), with one (1/6) neutral response (score = 5).

### 3.2. Study 2: Statewide Survey of MCHNs

MCHNs (*n* = 292) from 60 Local Government Areas in Victoria, Australia (*n* = 60/79, 76%) participated in the statewide survey.

On average, MCHNs, felt satisfied with the method they use to assess their clients’ emotional well-being (3.9 ± 0.9). Of the MCHNs who were most satisfied (scores 4–5), the majority completed the EPDS by paper and pencil (*n* = 169/200, 85%), verbally (*n* = 53/200, 27%), or electronically (*n* = 28/200, 14%), and a small proportion used the Whooley case finding questions (*n* = 5/200, 3%). A similar pattern was found for MCHNs who were least satisfied (scores 1–2), with 86% (*n* = 18/21) completing the EPDS via paper and pencil, 38% (8/21) verbally, 19% (*n* = 4/21) electronically, and 5% (*n* = 1/21) used the Whooley questions.

On average, MCHNs felt confident to very confident of what to do if a woman scored above the EPDS threshold (4.3 ± 0.7). The vast majority (231/275, 84%) responded that they would use a clinical decision support system if available to them.

From the open-ended questions, several general trends were identified for each question:

#### 3.2.1. Benefits to Using the PIRIMID System

1. Ensuring best practice

The most common potential benefit to using the PIRIMID system identified was the guidance and provision of a clear care pathway to ensure best practice, e.g., “Assistance with providing best management and support for mothers scoring high” (ID 174).

2. Efficiency

MCHNs reported that PIRIMID could improve convenience and efficiency, e.g., “Less time to complete… easy to access and complete and to either print off or email to GP” (ID 20), facilitate referrals, e.g., “Anything that can get to a GP is a good thing as they are historically very difficult to communicate with re any concerns due to their busy workload, rarely look at hard copy referrals, receptionists are their gate keepers and don’t put through calls. This would be a great step to providing better communication between our services” (ID 214), and support consistency across services, e.g., “Uniform approach across the MCH service, consistency in recommended treatment” (ID 230).

#### 3.2.2. Barriers to Using the PIRIMID System

1. Time constraints

Many MCHNs identified a lack of time as the major barrier, e.g., “We would need more time within consult to attend to this effectively” (ID 186).

2. Technical barriers

Many of the MCHNs also identified technical barriers, e.g., “Issues with internet access if the connection goes down” (ID 14), as well as reliable connectivity, e.g., “Often internet access is poor where I work … so if I depend on this method, it may not work on the computer” (ID 223). In addition, a lack of computer literacy and access to IT support were also identified as potential barriers, e.g., “If it is difficult or I didn’t have enough support to do it” (ID 180) and “I have experienced a lack of digital support for online issues in the past” (ID 146).

#### 3.2.3. What Makes It Possible/Easier to Use in Practice

1. Easy access such as integration with current systems

MCHNs reported that using PIRIMID in their routine practice would be facilitated by easy access, e.g., “Easily accessible and simple to use and timely to complete” (ID 67), and/or integration into existing system, e.g., “The questionnaire would populate automatically in CDIS [Child Development Information System]” (ID 125).

2. Sufficient time and training

MCHNs also identified the need for additional consultation time allocation and adequate training to facilitate use the system, e.g., “Increase the time allowed for each MCH KAS appointment” (ID 153) and “Ensure we have proper training to use this” (ID 63).

#### 3.2.4. How Screening Mothers for Depression and Anxiety Could Be Improved

1. Sufficient time

Again, sufficient time for the consultation was identified as a key factor, e.g., “Need to be given adequate time for discussion/implementation/referral making” (ID 327).

2. Timing and frequency

MCHNs expressed a preference for screening at different time points than current routine care and for repeated screening during the perinatal period, e.g., “I prefer to do routine EPDS at 8 weeks and again at 12 months” (ID 159). They also suggested alternate approaches to current paper and pencil screening, such as electronic methods or integration into the child health record, e.g., “I like to email the link to the woman, and they can fill it in on their own time” (ID 226).

3. Referral pathways

MCHNs highlighted the need for improvements to referral pathways to access appropriate services, e.g., “I think the screening tool that we use is fine—it’s the referral pathway and access to support services that is the problem” (ID 279).

## 4. Discussion

This study developed and pilot tested the PIRIMID system: an integrated e-screening and clinical decision support system that advances existing screening algorithm-based tools by guiding MCHNs to develop individualised management plans with specific referral pathways for women at risk of PND. If replicated in a fully powered study, the findings suggest the PIRIMID system may be a feasible and acceptable tool to assist MCHNs.

### 4.1. Rates of Referral and Treatment Uptake

There was a trend in favour of higher referral rates in the PIRIMID condition. This is consistent with previous research that has shown increases in rates of referral for treatment using a variety of approaches, including universal screening [11], standardised screening approaches that include a referral algorithm [17] and intensive systems of care with multidisciplinary dedicated teams [19]. Despite the trend for higher referral rates in the PIRIMID condition, treatment uptake appeared similar across conditions. Given the modest sample size and attrition in the study, and that much of the sample had minimal depression, anxiety and stress symptoms, an adequately powered RCT is needed to explore the impact of PIRIMID on treatment uptake further. This is important as treatment uptake following a positive screen for perinatal depression is typically low [12,14], and untreated PND not only adversely affects the woman, but also her family, and is associated with long-term negative outcomes for infants [8].

### 4.2. Depression, Anxiety and Stress

There was a suggestion that depression and anxiety symptom levels were reduced in the PIRIMID condition compared to the control condition. The proportion of cases with ‘moderate to severe’ symptoms numerically decreased between the 4-week KAS visit and 3-months post-birth follow-up, while the proportion in the Routine care condition in each study period numerically increased or did not change. This must be interpreted with care due to the small sample size. It is possible that the women in the PIRIMID condition sought help in ways that were not measured in this study (e.g., from partner, family or friends). The important role of social support in recovery from PND is evident in the literature [36]. It is also possible that the conversation PIRIMID MCHNs had with the women to develop a formulation and individualised management plan provided some benefits or impetus for change. Future research is needed to further explore this finding.

### 4.3. Acceptability of the PIRIMID System

Consumer feedback was sought from women and MCHNs at all phases of this project, contributing to the co-design and modifications of PIRIMID and facilitating improved implementation. MCHNs provided extensive feedback on operational barriers and content usability. The PIRIMID system was found to be acceptable and helpful to both MCHNs and women. Women in the PIRIMID condition rated their emotional health assessment on par with Routine care. MCHNs indicated that the PIRIMID system could be easily integrated into existing clinical systems. The majority of MCHNs surveyed in the state-wide survey indicated they would use PIRIMID if available to them and highlighted its potential to promote best practice through clear guidance, defined care pathways, and improved efficiency.

### 4.4. Screening Rates

Screening rates were high with almost all women screened with the EPDS and many also screened with the Whooley questions. Assessment of psychosocial risk factors for PND is recommended in the Australian Clinical Practice Guidelines [26] as part of routine screening. During study period 2, a large reduction was observed in the frequency of psychosocial risk factor assessments once PIRIMID MCHNs transitioned back to Routine care. This suggests that the integrated e-screening and PIRIMID system was needed to maintain a standard of emotional health assessment that is consistent with Clinical Practice Guidelines, and that education and training alone might not be sufficient to maintain high completion levels.

### 4.5. Future Research

The feedback obtained from women and MCHNs and the pilot RCT outcomes have informed improvements to the PIRIMID system and design of a fully powered cluster RCT (c-RCT). Changes and updates include:

(1) Version 2 of PIRIMID (used in the pilot RCT) has been updated and improved to version 3, as described in the methods, with a view to enhancing system usability.
(2) Training to use PIRIMID has been completely revamped and a digital training package developed that can be completed at the user’s convenience.
(3) Data collection and follow-up processes were revised to increase automation.
(4) The emotional health assessment has been moved to the 8-week KAS visit. This allows MCHNs to introduce the study at the 4-week KAS visit.
(5) Lack of time was an important factor repeatedly identified by MCHNs, so participating services have allocated an additional 15 min to 8-week KAS visits for study MCHNs.
(6) An option to complete e-screening via a link sent to the woman’s mobile device prior to her KAS visit has been added.
(7) Inclusion of a health economic evaluation to assess cost-effectiveness of the PIRIMID system.

### 4.6. Limitations

Whilst this study had many positives, there are also limitations that should be noted. The proportion of women who completed questionnaires in study period 1 was low. This may potentially introduce bias if participants who completed questionnaires differed from those who did not. However, improvements to data collection processes were made, including collecting data online and the option to complete questionnaires by telephone, so that completion rates in study period 2 were higher. It was also difficult to collect log data from MCHNs, and missing data was high (e.g., referral rates and duration of KAS visits). In addition, the COVID-19 pandemic impacted recruitment of both MCHNs and postnatal women, so sample size targets were not met, limiting statistical power. The small sample size and low symptom severity in this study limited its ability to provide conclusive evidence regarding clinical outcomes. Given the modest sample size and missing data, cautious interpretation of results is required and replication in an adequately powered trial is warranted.

As in almost every RCT, the generalisability of results needs consideration, as the study sample comprised only women willing to participate in research, again potentially introducing a selection bias. In addition, non-English speaking women were not included limiting generalisability of results. The baseline characteristics of women were relatively similar across the conditions; however, there appeared to be fewer Australian-born women in the PIRIMID condition. This difference may have influenced outcomes, as cultural differences can affect help-seeking and engagement, and should be considered when interpreting results. The study was also conducted in a relatively restricted geographic area in Melbourne, Victoria, Australia. As fathers were not included, further research is required to determine whether PIRIMID would need adaptation for use with fathers. Similarly, as PIRIMID was used at the 4-week KAS visit, future research could explore the utility and adaptations necessary for use in the immediate postnatal period (i.e., prior to 4 weeks postpartum), later in the postnatal period (e.g., at 8 weeks postpartum), and even during pregnancy.

As MCHNs were aware of their allocated intervention, this may have influenced their behaviour (Hawthorne effect), also potentially impacting generalisability. In addition, due to the partial crossover design, MCHNs who had used PIRIMID in study period 1 may have continued to apply elements when switching to Routine care in study period 2, potentially introducing carryover effects, making the Routine care condition in study period 2 similar to the PIRIMID condition and influencing comparison between the conditions. However, as the results showed a large reduction in the frequency of assessment of psychosocial risk factors once PIRIMID MCHNs transitioned to Routine care, these results suggest that the PIRIMID system may be needed to maintain a standard of emotional health assessment consistent with guidelines. Finally, because a single coder analysed the text responses from the statewide survey, there is a potential for bias in these results.

Nevertheless, the data obtained from this pilot study has been essential for exploring the feasibility and acceptability of the PIRIMID system and informing a larger c-RCT, which will provide more definitive results on the efficacy of PIRIMID. The changes described above for the planned fully powered c-RCT were implemented to enhance the PIRIMID system and to improve participant recruitment and retention.

## 5. Conclusions

This study made significant efforts to address an important area of research, the low uptake of treatment for postnatal depression. A clinical decision support system, PIRIMID, was developed and pilot tested to improve treatment uptake. If replicated in an adequately powered study, these findings suggest that PIRIMID may be a feasible tool to assist MCHNs to develop individualised management plans for women at risk of postnatal depression. With consumer feedback, the PIRIMID system has been updated for evaluation in a fully powered cluster RCT to explore the effectiveness of the system.

## Figures and Tables

**Table 1:** Outcomes and data collection timepoints.

	Data Collected from Women		Data Collected from MCHNs	
	4-Week KAS Visit (Baseline)	3-Months Post-Birth (Follow-Up)	4-Week Log	Study-End Feedback
**Maternal outcomes**				
Referral rates			X	
Treatment uptake	X	X		
Depression, anxiety and stress symptoms	X	X		
Depressive disorder		X		
Acceptability to women	X			
Participant demographics	X			
**MCHN outcomes**				
Screening rates			X	
Staff time			X	X (PIRIMID MCHNs only)
Acceptability to MCHNs				X (PIRIMID MCHNs only)

KAS = Key Age and Stage; MCHN = Maternal and Child Health Nurse; X = data collected at the specified timepoint.

**Table 2:** Participant characteristics and cluster sizes.

	PIRIMID Followed by Routine Care		Routine care Followed by Routine Care	
	Period 1: PIRIMID System (*n* = 52)	Period 2: Routine Care (*n* = 42)	Period 1: Routine Care (*n* = 57)	Period 2: Routine Care (*n* = 78)
**Maternal characteristics**				
Age (years), mean (SD)	31.8 (4.6)	31.3 (3.9)	31.8 (4.1)	32.4 (4.1)
Marital status, *n*/N (%)				
Married/living with partner	51/52 (98%)	38/40 (91%)	57/57 (100%)	73/75 (94%)
No partner	1/52 (2%)	2/40 (5%)	0	2/75 (3%)
Born in Australia, *n*/N (%)	29/52 (56%)	31/42 (74%)	44/57 (77%)	63/77 (82%)
Highest education, *n*/N (%)				
Certificate level or less	15/52 (29%)	13/42 (31%)	11/57 (19%)	19/78 (24%)
Advanced Diploma/ Diploma	10/52 (19%)	7/42 (17%)	12/57 (21%)	12/78 (15%)
Undergraduate degree	9/52 (17%)	14/42 (33%)	11/57 (19%)	22/78 (28%)
Postgraduate degree	15/52 (29%)	7/42 (17%)	20/57 (35%)	24/78 (31%)
Other	3/52 (6%)	1/42 (2%)	3/57 (5%)	1/78 (1%)
Income, *n*/N (%)				
Up to AUD80,000	17/51 (33%)	6/42 (14%)	16/57 (28%)	14/78 (18%)
Greater than AUD80,001	31/51 (61%)	28/42 (67%)	30/57 (53%)	52/78 (67%)
Do not wish to divulge	3/51 (6%)	8/42 (19%)	11/57 (19%)	12/78 (15%)
Currently receiving counselling or psychological therapy, *n*/N (%)				
No	50/52 (96%)	38/42 (91%)	51/57 (90%)	73/78 (94%)
Yes	2/52 (4%)	4/42 (10%)	6/57 (11%)	5/78 (6%)
Once	0	0	1/6 (17%)	0
Occasionally	2/2 (100%)	2/4 (50%)	2/6 (33%)	2/5 (40%)
Regularly	0	2/4 (50%)	3/6 (50%)	3/5 (60%)
Currently taking antidepressants or medication for anxiety, *n*/N (%)	2/52 (4%)	3/42 (7%)	5/57 (9%)	2/78 (3%)
EPDS score, median (IQR)	6 (3–10)	6 (3–9)	4 (3–8)	6 (3–9)
EPDS score, *n*/N (%)				
No/minimal depression (0–9)	38/52 (73%)	32/42 (76%)	49/55 (89%)	63/77 (82%)
Mild depression (10–12)	9/52 (17%)	6/42 (14%)	2/55 (4%)	8/77 (10%)
Moderate to severe depression (13+)	5/52 (10%)	4/42 (10%)	4/55 (7%)	6/77 (8%)
Number of children, *n*/N (%)				
One	23/52 (44%)	25/42 (60%)	30/56 (53%)	45/78 (58%)
Two	22/52 (42%)	14/42 (33%)	21/56 (38%)	23/78 (30%)
Three or more	7/52 (14%)	3/42 (7%)	5/56 (9%)	10/78 (13%)
**Cluster sizes,** median (range)	7 (5–21)	5 (0–17)	9 (0–18)	12 (0–28)

PIRIMID = Perinatal Identification, Referral and Integrated Management for Improving Depression; SD = Standard deviation; IQR = Interquartile range (25th to 75th percentiles); EPDS = Edinburgh Postnatal Depression Scale. Note. Only non-missing responses (valid percentages) are reported. Percentages may not equal to 100% due to rounding.

**Table 3:** Maternal outcomes at 4-week KAS visit and 3-month follow-up.

	PIRIMID Followed by Routine Care		Routine Care Followed by Routine Care	
	Period 1: PIRIMID System (*n* = 52)	Period 2: Routine Care (*n* = 42)	Period 1: Routine Care (*n* = 57)	Period 2: Routine Care (*n* = 78)
Referred by MCHN, *n*/N (%) [95% CI]	4/22 (18%) [5–40%]	6/41 (15%) [6–29%]	3/29 (10%) [2–27%]	7/52 (14%) [6–26%]
Treatment uptake, *n*/N (%)				
Between birth and 4- week KAS visit	15/44 (34%)	15/42 (36%)	18/53 (34%)	26/72 (36%)
Between 4-week KAS visit and 3-months post-birth ^a^	5/19 (26%)	8/34 (24%)	10/32 (31%)	16/62 (26%)
Depression, median (IQR)				
At 4-week KAS visit	1 (0–4)	2 (0–6)	1 (0–4)	2 (0–6)
At 3-months post-birth	2 (0–6)	1 (0–8)	2 (0–8)	2 (0–6)
Moderate to severe depression, *n*/N (%)				
At 4-week KAS visit	4/52 (8%)	2/40 (5%)	5/56 (9%)	5/72 (7%)
At 3-months post-birth	1/19 (5%)	3/34 (9%)	6/32 (19%)	4/61 (7%)
Anxiety, median (IQR)				
At 4-week KAS visit	2 (0–6)	2 (0–6)	2 (0–6)	2 (0–4)
At 3-months post-birth	2 (0–8)	0 (0–4)	2 (0–4)	2 (0–4)
Moderate to severe anxiety, *n*/N (%)				
At 4-week KAS visit	6/52 (12%)	5/40 (13%)	8/55 (15%)	7/72 (10%)
At 3-months post-birth	0	3/33 (9%)	3/32 (9%)	5/61 (8%)
Stress, median (IQR)				
At 4-week KAS visit	7 (2–12)	8 (4–14)	6 (2–14)	10 (4–14)
At 3-months post-birth	6 (2–10)	6 (0–14)	8 (6–15)	10 (4–14)
Moderate to severe stress, *n*/N (%)				
At 4-week KAS visit	4/52 (8%)	2/39 (5%)	6/56 (11%)	6/72 (8%)
At 3-months post-birth	2/19 (11%)	4/33 (12%)	5/27 (16%)	7/58 (12%)
Depressive disorder diagnosis at 3-months post-birth, *n*/N (%)	2/33 (6%)	3/36 (8%)	6/40 (15%)	3/58 (5%)
Emotional health assessment, median (IQR)				
Helpful	9 (8–10) *n* = 51	9 (6–10) *n* = 42	10 (8–10) *n* = 57	8 (6–10) *n* = 73
Comfortable	9 (8–10) *n* = 52	9 (8–10) *n* = 42	10 (9–10) *n* = 57	9 (7–10) *n* = 73

^a^ Treatment obtained from a psychologist/counsellor or doctor. For a breakdown of help sought from other sources, see Supplementary Materials Table S1. PIRIMID = Perinatal Identification, Referral and Integrated Management for Improving Depression; MCHN = Maternal and Child Health Nurse; IQR = Interquartile range (25th to 75th percentile); KAS = Key Age and Stage. Note. Only non-missing responses (valid percentages) are reported.

**Table 4:** MCHN screening rates and duration of 4-week KAS visit.

	PIRIMID Followed by Routine Care		Routine Care Followed by Routine Care	
	Period 1: PIRIMID System (*n* = 52)	Period 2: Routine Care (*n* = 42)	Period 1: Routine Care (*n* = 57)	Period 2: Routine Care (*n* = 78)
Screened				
EPDS, *n*/N (%)	52/52 (100%)	42/42 (100%)	57/57 (100%)	77/78 (99%)
Whooley questions, *n*/N (%)	51/52 (98%)	13/40 (33%)	6/28 (21%)	13/52 (25%)
Psychosocial risk factors, *n*/N (%)	52/52 (100%)	18/40 (45%)	25/27 (93%)	43/52 (83%)
Duration of 4-week KAS visit, median (range)	45 (45–60) *n* = 21	45 (30–60) *n* = 41	45 (40–60) *n* = 29	45 (30–60) *n* = 52

PIRIMID = Perinatal Identification, Referral and Integrated Management for Improving Depression; EPDS = Edinburgh Postnatal Depression Scale; KAS = Key Age and Stage. Note. Only non-missing responses (valid percentages) are reported.

## Data Availability

The data from this study are not publicly available, as participants did not provide consent for their data to be shared in a public repository. De-identified data may be available from the corresponding author upon reasonable request and subject to approval by the Human Research Ethics Committee of Austin Health. Access will be granted on a case-by-case basis in accordance with institutional and ethical guidelines.

## Supplementary Materials

The following supporting information can be downloaded at https://www.mdpi.com/article/10.3390/healthcare13202578/s1. Table S1: Services or programs used to manage mood between the 4-week KAS visit and 3-months post-birth.
