# Supplementary material for: Perinatal Identification, Referral, and Integrated Management for Improving Depression: Development, Feasibility and Pilot Randomised Controlled Trial of the PIRIMID System
Source: Healthcare (Basel). 2025 Oct 14;13(20):2578. doi: 10.3390/healthcare13202578 (PMC12564491; doi:10.3390/healthcare13202578)
Supplement: Supplementary file 1 [file healthcare-13-02578-s001.zip › Table S1.pdf]

SUPPLEMENTARY TABLE

Table S1: Services or programs used to manage mood between the 4-week KAS visit and 3-months post-birth

	**PIRIMID followed by Routine care**		**Routine care followed by Routine care**	
	**Period 1: PIRIMID system (n=52)**	**Period 2: Routine care (n=42)**	**Period 1: Routine care (n=57)**	**Period 2: Routine care (n=78)**
Sought help, n/N (%)				
Doctor	5/19 (26%)	8/34 (24%)	6/32 (19%)	10/62 (16%)
Psychologist/counsellor	2/19 (11%)	4/34 (12%)	4/32 (13%)	6/62 (10%)
Antidepressant medication	2/19 (11%)	1/34 (3%)	3/32 (9%)	3/62 (5%)
Internet-based treatment program	1/19 (5%)	1/34 (3%)	0/32 (0%)	2/62 (3%)
Maternal and Child Health Nurse	6/19 (32%)	12/34 (35%)	15/32 (47%)	18/62 (29%)
Self-help book	0/19 (0%)	4/34 (12%)	2/32 (6%)	6/62 (10%)
Telephone support helpline	1/19 (5%)	1/34 (3%)	1/32 (3%)	3/62 (5%)
Websites	1/19 (5%)	3/34 (9%)	3/32 (9%)	7/62 (11%)

Only non-missing responses (valid percentages) are reported. Percentages may not equal to 100% due to rounding.
